# Continuous Glucose Monitoring in Patients Undergoing Extracorporeal Ventricular Assist Therapy

**DOI:** 10.1371/journal.pone.0148778

**Published:** 2016-03-10

**Authors:** Antje Gottschalk, Henryk A. Welp, Laura Leser, Christian Lanckohr, Carola Wempe, Björn Ellger

**Affiliations:** 1 Department of Anesthesiology, Intensive Care and Pain Medicine, University Hospital Münster, Münster, Germany; 2 Department of Cardiothoracic Surgery, Division of Cardiac Surgery, University Hospital Münster, Münster, Germany; Azienda Ospedaliero-Universitaria Careggi, ITALY

## Abstract

**Background:**

Dysregulations of blood glucose (BG) are associated with adverse outcome in critical illness; controlling BG to target appears to improve outcome. Since BG-control is challenging in daily intensive care practice BG-control remains poor especially in patients with rapidly fluctuating BG. To improve BG-control and to avoid deleterious hypoglycemia, automated online-measurement tools are advocated. We thus evaluated the point-accuracy of the subcutaneous Sentrino^®^ Continuous Glucose Monitoring System (CGM, Medtronic Diabetes, Northridge, California) in patients undergoing extracorporeal cardiac life support (ECLS) for cardiogenic shock.

**Methods:**

Management of BG was performed according to institute’s standard aiming at BG-levels between 100–145 mg/dl. CGM-values were recorded without taking measures into therapeutic account. Point-accuracy in comparison to intermittent BG-measurement by the ABL-blood-gas analyzer was determined.

**Results:**

CGM (n = 25 patients) correlated significantly with ABL-values (r = 0.733, p<0.001). Mean error from standard was 15.0 mg/dl (11.9%). 44.2% of the readings were outside a 15% range around ABL-values. In one of 635 paired data-points, ABL revealed hypoglycemia (BG 32 mg/dl) whereas CGM did not show hypoglycemic values (132mg/dl).

**Conclusions:**

CGM reveals minimally invasive BG-values in critically ill adults with dynamically impaired tissue perfusion. Because of potential deviations from standard, CGM-readings must be interpreted with caution in specific ICU-populations.

## Introduction

Dysregulations of blood glucose levels (BG) are frequent among critically ill patients with and without a history of diabetes mellitus. The notion to interpret this stress induced dysglycemia as beneficial does not hold true since not only hyperglycemia but also hypoglycemia and fluctuations of glycemia are associated with adverse outcome in critically ill patients [1–6]. Multiple clinical trials and experimental studies in the last decade reported attempts to control dysglycemia by titrated insulin infusion. The concept is meanwhile well supported by evidence and mechanisms are largely understood [7]. However, beneficial effects on mortality and morbidity could not be confirmed in multi center trials [8–10]. The most convincing explanation is that in the daily clinical setting controlling glycemia is complicated by issues like quality of the algorithms, care-givers work-load and training, measuring technologies and route of insulin administration as well as poor protocol adherence to insulin titration protocols. Hence BG-control remained poor in everyday life and, especially when normoglycemia is the target, the risks of hypoglycemia and fluctuations of BG are amplified, both causally linked to increased mortality [5; 6; 11] and permanent neurocognitive impairment [12; 13]. Insufficient management of glycemia might thus completely offset any potential clinical benefit of avoiding severe hyperglycemia. Moreover, different patient groups appear to have unlike optimal BG-corridors during critical illness or in the perioperative period [9;14]. Therefore, strict glycemic control (target 80–110 mg/dl) is currently not recommended for critically ill patients.

Various algorithms have been implemented targeting improved control of dysglycemia. Since patients very individually and in a disease related manner react to interventions in glucose metabolism, efficacy and safety are not easy to obtain [15]. Especially when BG is strictly controlled to normoglycemia by intermittent measures, hypoglycemic episodes occurring in between measurements cannot be excluded. Recently, sophisticated software guided solutions [16] or even learning algorithms [17] promised acceptable performance. However, the protocols demand nurse attention and time to measure BG, thus BG-control is considered an additional burden in everyday life resulting in poor protocol compliance [18]. Developing a system to measure BG automatically and adapt insulin dose accordingly can be advocated. Steps towards a more or less closed-loop system were indeed promising [19], precluded by the on-line BG-measurement technology. Some measurement-devices were developed and are currently validated in the critically ill population [20; 21]. One of them, the subcutaneous continuous glucose monitoring (CGM, Medtronic Sentrino^®^, Medtronic Inc., Northridge, CAL/USA) provides minimal invasive online BG-values and prior studies suggested an acceptable accuracy [22]. Moreover, when patients are monitored by CGM, the incidence of hypoglycemia might be lowered [23].

When sampling in the subcutaneous, interstitial compartment in critically ill patients, rapid and profound alterations of microcirculation must be taken into account. A reduced substrate exchange of the interstitial fluid by changes in macro- and microcirculatory perfusion and capillary integrity could change glucose levels in an unpredictable manner. Especially, the sickest patients carry the highest risk of affected microperfusion and as well the highest risk for profound hyperglycemia and rapidly developing hypoglycemia. Patients undergoing extracorporeal cardiac life support (ECLS) for cardiogenic shock can be expected to be prone to such severely affected perfusion and a high risk of hypoglycemia; however, misguidance of therapy by erroneous measurement readings is deleterious especially in this cohort.

This study prospectively evaluated the performance and safety of a minimal invasive online measurement tool for blood glucose in a patient population who underwent ECLS therapy for cardiogenic shock.

## Methods

### Primary study hypothesis

In critically ill patients undergoing extracorporeal life support the CGM-sensor Medtronic Sentrino^®^ provides continuous accurate surrogates for blood glucose levels as compared to standard of care. The tool thus helps to reduce the incidence of hypoglycemic episodes in this group of severely ill patients.

### Study design

With approval of the Ethics Committee of the Medical Council of Westphalia-Lippe and the Medical School of the University of Muenster (Number of the ethical approval: 2012-645-f-S) we prospectively included patients undergoing a) rescue ECLS for cardiogenic shock and/or b) left ventricular assist device (LVAD) implantation into our prospective, observational study. Written informed consent was obtained either from the patients prior to the procedure or next relatives in case of patient`s inability to wave consent. Within 24 hours after surgery, we placed and secured a CGM-sensor on the anterior thigh according to the user`s manual. Calibrations were performed initially according to the manufacturer`s guidelines and consecutively in 8 hours intervals. An investigational period of 72 hours was aimed for with each sensor. Glucose-readings obtained from the sensor were automatically recorded.

Treatment of the patients and management of BG was performed according to the institute’s standard operating procedures aiming at a BG between 100 and 145 mg/dl, so far sensor values were not taken into therapeutic consideration. Since nurses were not blinded to GCM values, event-related reactions to device values or alarms, followed by confirming BG measurements, could not be excluded; these reactions could not be traced in this study. For BG-control we exclusively used arterial BG values derived from an ABL-blood-gas analyzer (ABL 800, Radiometer, Germany). Quality control of ABL was performed according to the user`s manual by a certified technician.

### Glucose sensor

The Medtronic Sentrino^®^ CGM-device consists of a monitor recording actual glucose value and trend over time, connecting cable and the sensor. This sensor is made of a self-adhesive plate and two flexible sensor stripes that are placed into subcutaneous tissue with removable insertion needles, preferably in the anterior thigh or the abdomen. Glucose concentration in the tissue fluid is therefore continuously measured by glucose-oxidase method; after calibration with the standard method, via a mathematical model, BG is calculated and in 1 minute intervals a new glucose-value is displayed. Three initial calibration-measurements with a standard method and control-measurements every 8 hours are needed to calibrate the system upon system request.

The sensor is resistant to interference of drugs that are commonly used in ICU. The sensor is approved for running periods of up to 72 hours.

### Endpoints

We investigated the point-accuracy of the technology during critical tissue perfusion. As a parameter for point-accuracy, ISO-norm for Germany requires that measured values of a glucose monitoring device should vary ± 15% around the standard value. Current consensus statements advocate a stricter minimum standard for glucose meters to be used in critically ill patients: 98% of readings should be within a 12.5% range around a reference standard. The mean absolute relative difference should be <14%; values >18% are considered to represent poor accuracy [24]. Moreover, we report the results of the correlation analysis. Mind that these consensus-standards might not be fully applicable to CGM since, besides punctual values, CGM also provides trends; however for trend accuracy there is not yet an acceptable measure.

As well, the system should alarm whenever the signal is insufficient to generate a valid measure rather than providing doubtable values.

Furthermore, we recorded the incidence of hypoglycemic episodes that were recorded by CGM but were missed by intermittent measurements or vice versa. We recorded the duration of the durability of the sensor and any potential interference by physiological or therapeutic issues (e.g. hyperbilirubinemia). Finally we recorded any potential adverse events of the sensor like bleeding, bruising, skin irritation or infections.

### Statistical analysis

To test for two-sided sample equivalence, sample size was calculated as follows: Mean blood glucose values were estimated to be 135 mg/dl with a standard deviation of 30 mg/dl. This estimation was based on the years average of blood-glucose values on our ICU. Since aberrations of 15% are considered as poor accuracy a minimal sample size of 20 patients was necessary to detect a 15%-aberration with a power of 80%. We thus included a sample size of 25 patients to account for potential drop out.

Analyses were performed by SPSS (SPSS 20.0 (IBM, New York, NY). We used Pearsons Coefficient and Bland-Altman plot for accuracy analysis. Percentage of values outside a 12.5% frame around the standard method and mean deviation from the ABL-values were calculated. Additionally we provide a Clark-error grid analysis although it might be misleading in ICU-patients [24]. A p-value of < 0.05 was considered significant.

A supporting information data set is published as S1 Data; a more extensive data set is available upon request to Dr. Henryk Welp (henryk.welp@ukmuenster.de).

## Results

25 patients (19 male, 6 female) were included in our study. Demographic data of the study population are shown in Table 1. All patients received vasopressor agents (epinephrine, norepinephrine or vasopressin) at various doses during the study period. None received vasodilators during the study period.

**Table 1:** Demographic data.

	Median	Minimum	Maximum	Percentiles (25th, 50th, 75th)		
Age [years]	57	19	75	52	57	69
Height [cm]	175	153	198	169,5	175	180
Weight [kg]	90	64	120	78	90	105
SAPSII (admission)	49	6	109	36	49	75
SAPSII (study start)	75	29	109	59	75	88
APACHE (admission)	33	12	46	24,25	33	39,75
1st SOFA	14	3	19	8	14	15
SOFA (study start)	15	7	20	13	15	18
1st ROD	43,8	0,5	99,3	18,1	43,8	88,9
ROD (study start)	88,9	9,7	99,3	66,1	88,9	96,1

**Fig 1:**
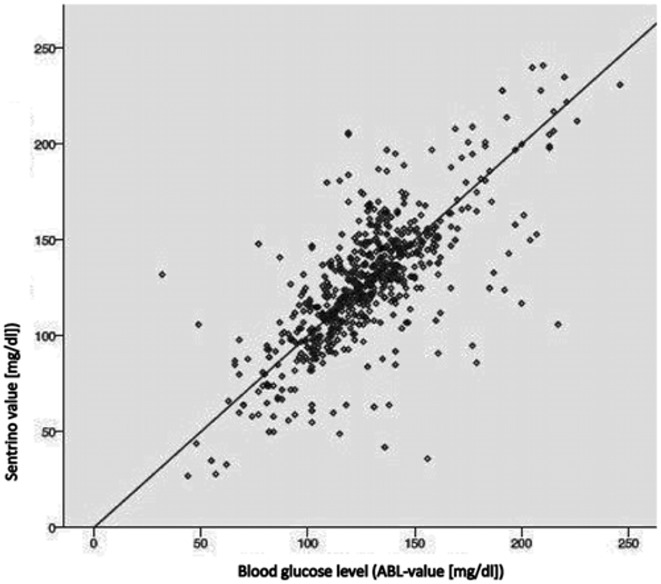
Regression analysis, Pearson correlation: 0.733398, significance (2-tailed), p = 0.000.

**Fig 2:**
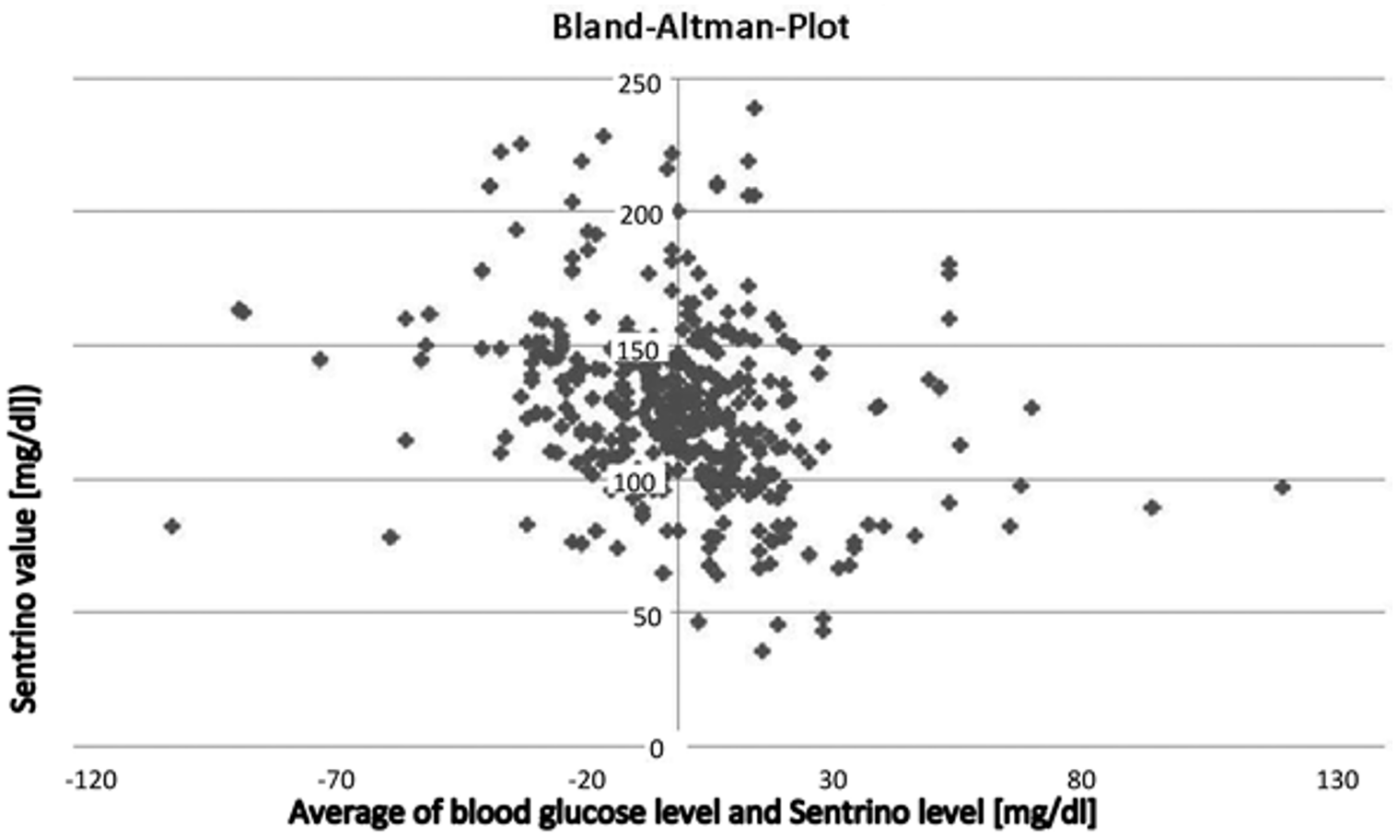
Bland-Altman-Plot.

**Fig 3:**
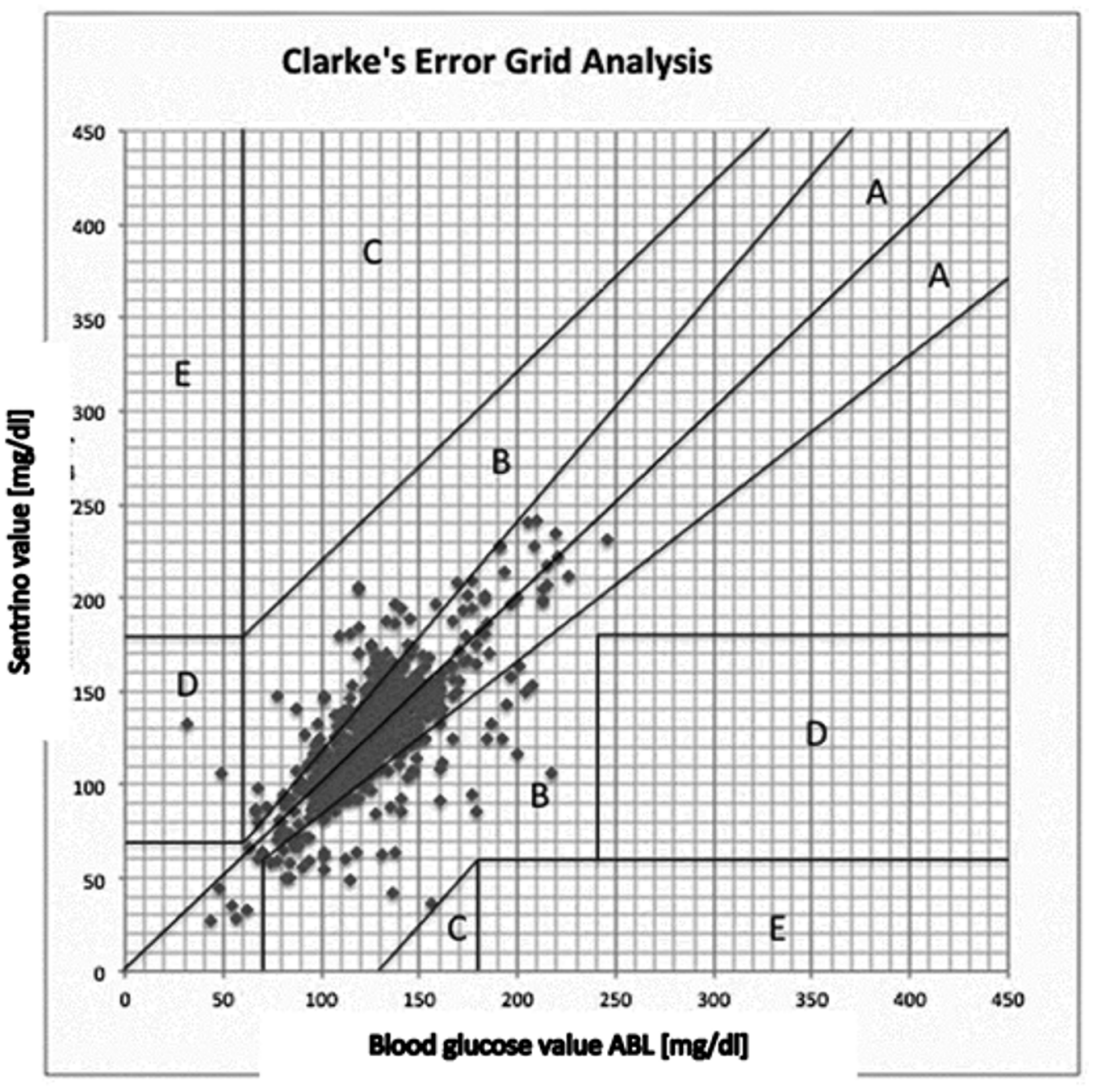
Clarke Error Grid Analysis. Zone A: Clinically accurate, Zone B: Benign, Zone C: Overcorrected, Zone D: Failure to detect, Zone E: Erroneus.

In all patients the Sentrino^®^ sensor was inserted successfully and therefore for all patients initial measurement were obtained. We obtained 635 comparing glucose-results (ABL-value and time matched CGM-value), the values ranged from 27 mg/dl to 246 mg/dl. As primary end point, 55.8% of the CGM-values were within 15% as compared to the standard method; 49.1% of the CGM-values were within a 12.5% range around the ABL-values. The median error from standard was 15.0 mg/dl (11.9%). The CGM-results correlated significantly (r = 0.733, p < 0.001) (Fig 1), however Bland-Altman (Fig 2) revealed relevant deviations. CGM-values revealed no systematic off-set (in mean 0.25 mg/dl higher as standard method). Clarke`s error grid (Fig 3) revealed no values in zone E, 2 values were in zone D which means that two relevant and potentially dangerous hypoglycemic values were not detected by Sentrino^®^.

We recorded 73703 Sentrino^®^ -values of which 671 were below 60 mg/dl (0.9%). In the 635 ABL-values, we detected 6 values below 60 mg/dl (0.9%). In one case, ABL revealed BG of 32 mg/dl whereas the Sentrino^®^ -system did not reveal hypoglycemic values at the time point or in a 60 minutes window around the ABL-measurement (CGM reading 132 mg/dl at the same time point). In the technical readout no technical alarm could be identified, there was no clue of bad signal quality or malfunction of the tool.

In 2 patients CGM did not reveal any stable measurement-signal and the system showed the system alarm for poor sensor signal quality. These two patients were consecutively diagnosed with a complete thrombosis of the arterial leg vessels. Even bilirubin levels above 40 mg/dl did not affect the signal. In 13 of the 25 patients the sensor completed a full 72 hours run. Causes for an incomplete 72-hour measurement were clinical reasons, e.g. surgical intervention, MRT or death. No malfunction of the device occurred and no adverse events of the sensor like dislocation, bruising, bleeding, skin affections or signs of infection were recorded.

Median BG on our ICU was 137 mg/dl (+/- 36.5 mg/dl standard-deviation); 0.3 ‰ of BG-values were below 40 mg/dl.

## Discussion

In this prospective observational study in critically ill adults undergoing cardiac and/or cardiopulmonary assist device implantation, the subcutaneous on-line glucose measuring device Sentrino^®^ revealed glucose values that were closely correlated to standard of care method. Accuracy, however, did not meet the strict ISO-norm criteria for Germany Nevertheless, the continuous data provide more comprehensive glucose trend information that put point-accuracy into perspective.

Especially in the acute phase of severe critical illness severe hyperglycemia occurs and BG values fluctuate fast. Moreover, especially when sickness is aggravated by liver dysfunction, the risk of hypoglycemia is amplified. These endocrine dysregulations are all independent predictors of poor prognosis and as such deserve therapeutic attention [6; 14]. Attempts to control dysglycemia by titrating insulin-infusion to target normoglycemia revealed controversial results [7]. Especially multi-center trials showed that controlling BG in everyday life is challenging for several reasons and a liberal BG-target (e.g. below 180 mg/dl) appears safer and superior to very tight glucose control (target 80–110 mg/dl) [25]. One reason is that in real life protocols for glucose control are neglectfully followed and frequent glucose control is considered both an additional burden and a nuisance in patient care [18]. So side-effects, especially hypoglycemia, occur frequently and offset potential benefits [11]. Consequently, current guidelines no longer recommend tight glycemic control [26]. Notably, even when guidelines are followed and a liberal glucose target is aimed at, up to 3% of patients are expected to experience at least one episode of hypoglycemia with all its harmful effects [7]. This leads to the quintessential realization that BG control in critically ill patients is a rather complicated therapeutic intervention that is vitally dependent on proper monitoring and adherence to predefined protocols. This defines the need to improve BG-control in everyday life. Since workload for caregivers and availability of educated staff is limited by financial restriction, it is tempting to develop technical tools to save human resources. The measurement of glucose levels appears reasonable in both blood and interstitial fluid. The latter appears tempting since no vascular access with infectious hazards or clotting is needed and experience from diabetes care is available. Indeed, recently a subcutaneous method was evaluated in a cohort of critically ill patients. Accuracy and the correlation with standard of care measurements appeared reasonable [21]. Two recent studies in adult critically ill patients admitted after major abdominal surgery [27] and after cardiac surgery [28] resemble the results. One has to keep in mind that these studies included patients with less severity of illness as it for example can be concluded from reported SAPS II scores (e.g. [27]: 35; our data: 75 upon study start). In our study, we largely underlined published data with an improved software version and showed good estimation of blood glucose by the sensor in a wide range of BG. However, in Bland-Altman analysis, we recognized relevant mistakes of CGM as compared to standard care. Moreover, when the very strict criteria of current ISO-norm for point-accuracy with glucometer devices are taken as a basis, with more than 40% of the values outside a 15% range around the reading given by the standard method, the performance in this special cohort of patients appears rather poor. Current consensus recommendations for ICU-patients promote that 98% of readings are within a 12.5% range around the true value, performance is considered poor when mean difference is above 18%. Keeping in mind that our cohort of patients represents the most critical ICU population, although CGM does clearly not fully meet the strict criteria, a mean difference of 15% from standard of care as shown in our data still appears acceptable [24]. Indeed, as written in the product handout, Sentrino^®^-values have to be confirmed by a standard of care method, especially in critical medical conditions. Our finding underlines our concerns that rose from the results of previous trials that the published very good results in point-accuracy [22] might have been restricted to less severely affected patients. In circulatory situations signified by a profound impairment of microperfusion, like the ECLS-patients in our trial, subcutaneous sampling as a stand-alone method is clearly inappropriate, probably providing incorrect values that could lead to erroneous therapeutic decisions when insulin dosing is based on CGM-values only. Indeed, in two cases, as shown in the Clarke`s grid, hypoglycemic values would not have been detected that would otherwise have had an impact on glucose management. One has to keep in mind that this way of analyzing might not be suitable for critically ill and to compare discontinuous measurements to continuous methods as extensively discussed by expert commission [4].

Besides the mentioned critical deviation from standard-care values, CGM occasionally did not provide any readings during most severe hypoperfusion but an alarm-message. The software-algorithms thus managed to prevent display of erroneous information. However, it failed to impede giving wrong estimates of BG that are intrinsic to the technique. This could most probably be a delay in the equilibration between glucose levels in the blood and in subcutaneous tissue.

Similar to bench-top experiments by the manufacturer we could not detect any relevant interference of commonly used drugs or medical afflictions with the sensor, even severe hyperbilirubinemia of above 40 mg/dl or profound anemia did not affect the measures.

Besides using CGM-values for BG-control algorithms, CGM might be applied as a safety-tool to detect hypoglycemia that would have been camouflaged within the measurement interval between two ABL-measures. In our cohort where CGM values were not taken into therapeutic consideration, we did not detect any episode of hypoglycemia with CGM that was not detected by our intermittent control. Since in our center glycemic control is well established targeting BG between 100 and 145 mg/dl with an incidence of hypoglycemia (BG below 40 mg/dl) of only 0.3‰ of all BG-measurements (<1% of the patients), this is not puzzling. We did not detect one rapidly developing hypoglycemia by CGM. This might again have been due to the relatively slow equilibration of BG and glucose concentration in the interstitial fluid in our patients that all had impaired microperfusion and inflammation induced edematous swelling of subcutaneous tissues. Since assessing microperfusion and it`s influence on subcutaneous measurement is anything but simple, we thus must be aware that in this most critical cohort, very rapid changes of BG might, if at all, be reflected with a delay by CGM. Although vasoactive medication might potentially play a role in this context, we resigned to perform a distinct analysis. Vasopressors are an intrinsic part of the treatment in our patient group anyway and must be considered as a consequence rather than the reason for the sickness and altered microperfusion. Conclusively, CGM in subcutaneous tissue will surely not be able to totally avoid hypoglycemia. This statement is largely supported by findings from a recent study where CGM reduced the incidence of hypoglycemia from 11.5% to 1.6% [22]. In this trial, BG-targets were below 110 mg/dl. Notably, in our institution the incidence of hypoglycemia without CGM, probably due to a higher glucose target, is lower as in the mentioned study. So detection of hypoglycemia in our small cohort is rather unlikely.

CGM might be used to decrease the frequency of glucose measures and thus, apart from safety considerations, reduce human work-load. Indeed, well conducted glucose control might be cost-beneficial [29]. Given that benefits of improved BG-control by CGM outweigh its costs, the intervention might be cost-beneficial. Whether this holds true in real life must be shown in future trials as well as benefits on morbidity and mortality.

Indeed, it is tempting to speculate that CGM might improve morbidity and mortality by improving all three domains of BG-control [19]. Our study was not designed to attack this question. It remains speculative that, although our protocol foresaw not to take GCM-value into therapeutic considerations, care givers might have been influenced to take a BG sample when GCM suggested a disadvantageous trend of glycemia and by this unintendedly improved BG control. As well, when narrow BG-corridors can be achieved by technical support, the unresolved issue that different patient groups might profit from distinct target corridors [14] can be attacked. However, a group from Austria showed that stratified CGM-values used to guide insulin therapy by a paper-based algorithm in analogy to intermittent BG measurement, glucose control did not improve [22; 30]. Solely using CGM as an alternative punctual measurement method does not do the job. Indeed, one important information that CGM can provide is the early detection and readout of a BG-trend. This information that complements punctual measurements was completely left out of consideration in published studies. Besides not taking BG-trends into account, the applied algorithm did not incorporate issues like adaptations in nutrition and individual sensitivity to insulin. A computerized self-learning algorithm that additionally considers trends in BG-levels and predicts glucose values—or at least most probable corridors—on individual basis would most probably improve BG control [17]. Semi-automated Insulin administrating devices that titrate insulin infusion depending on such a learning algorithm have been validated and have proven very good performance [1; 16; 31]. Combining automated measuring, controlled by intermittent calibrations to the standard of care BG-measurements, with a computerized learning algorithm and an automated insulin-infusion device would probably be the key to success. Indeed, recently such a closed-loop system with an intravascular sensor was introduced with promising results [19]. Since it provides reasonably valid and safe data, subcutaneous CGM by Sentrino^®^-technology would, in our view, be a minimal invasive partner in a closed-loop system avoiding the flaws and hazards of an intravascular access.

As a limitation of the device it has to be considered that in a high percentage of the patients the sensor was not functional for the designated 72 hours for several reasons. This largely resembles data from published studies that report a high percentage of sensors that had to be replaced before completing a 72 hours run (28% in [28]). In two patients no measurements at all were possible at the anterior thigh after the initial calibration because of obvious limb ischemia. In others the signal faded after some hours, most likely because of critical local perfusion as well. Additionally, due to the relatively small number of patients we can only conclude on the point-accuracy of the method. Since there is no valid measure for trend-accuracy we cannot discuss this issue conclusively. Moreover, conclusions on the clinical applicability in every-day life and Sentrino`s^®^ impact on patient`s outcome cannot be concluded from this trial.

In conclusion, although Sentrino^®^ CGM provides the features advocated in a consensus statement [24] for effective and safe continuous and automated BG-monitoring and was shown to be of good value in most critically ill patients, our results suggest that because of considerably high measurement errors the device is not suited as the sole monitoring device for blood glucose measurements in patients with exceedingly severe microcirculatory impairment due to mechanical circulatory support. It appears reasonable to handle the tool with caution and to regularly control glycemia by a standard method when the tool is applied to virtually all ICU-patient populations.

## Supporting Information

S1 Data supporting information data set. (XLSX)
